# In vitro blood compatibility evaluation method: incubating while rotating hemodialyzers filled with fresh human blood

**DOI:** 10.1007/s10047-020-01224-6

**Published:** 2020-11-16

**Authors:** Kinue Kamata, Yoshihiro Hatanaka, Hiromi Tanaka, Satoru Inoue, Yusuke Tokimizu, Sayuri Tanba, Yuki Kishikawa, Toshinori Koizumi

**Affiliations:** Medical Technology and Material Laboratory, Research and Business Development Division, Asahi Kasei Medical Co., LTD., 2-1, Samejima, Fuji-City, Shizuoka 416-8501 Japan

**Keywords:** Vitamin E-bonded polysulfone membrane, Blood compatibility, Antithrombotic property, Antioxidative property, Anti-inflammatory property

## Abstract

One of the often-used methods for in vitro evaluation of the blood compatibility of hemodialysis membranes is the circulation of human blood through a miniaturized hemodialyzer. The use of a rather small amount of human blood in its evaluation is one advantage of this method. However, because it is manufactured by a different process than actual ones, a miniaturized hemodialyzer membrane cannot always preserve the properties of actual hemodialyzers. To address this problem, we established a new experimental method that uses a relatively small amount of human blood and actual dialyzers. In this method, a test hemodialyzer and a control hemodialyzer filled with human blood obtained from the same donor is slowly rotated to prevent spontaneous blood cell sedimentation for 4 h at 37 °C. By use of this method, we were able to compare blood compatibility between a polysulfone (PS) membrane and a vitamin E (VE)-bonded PS membrane in terms of their relative antithrombotic, antioxidative, and anti-inflammatory properties. Consistent with many previous reports, the results clearly showed that compared with the PS membrane, VE-bonded PS membrane is more blood compatible. These findings suggest that our method is applicable, at least to in vitro blood compatibility evaluation of PS type dialysis membranes.

## Introduction

When the blood of a patient undergoing hemodialysis touches the hemodialysis membrane, which is an artificial material, it is generally known that the platelets are activated [1], and consequently induces platelet adhesion on the hemodialysis membrane surface accompanied by platelet–platelet interaction and platelet–leukocyte interaction, causing thrombus formation. Simultaneously, leukocytes are also activated promoting the production of oxygen radicals [2].

Many improvements in the materials of hemodialysis membranes have been made to improve blood compatibility so far [3]. Two evaluation method have been employed in studies to improve the blood compatibility of the materials. One is that using human blood and a miniaturized hemodialyzer [4], and the other is that using animal blood and actual hemodialyzers [5].

The former method can evaluate the blood compatibility, using a rather small amount of human blood. However, it has difficulty of setting the experimental conditions to clinical treatment. Also, it cannot evaluate an actual dialysis membrane, the properties of which are preserved as manufactured and employed in clinical use. Since preparing a miniaturized hemodialyzer process includes manually breaking down and reassembling, the chemical and physical properties of dialysis membrane can be different from that of the actual one.

On the other hand, the latter method can be easily used to set an experimental condition to clinical treatment. Also, it can evaluate an actual dialysis membrane. However, it cannot evaluate human blood compatibility. As pointed out in ISO 10993-4, possible effects on human blood are not always consistent with the results obtained from animal blood, which depends on the species.

To overcome the above-mentioned drawbacks of in vitro evaluation, we established a new experimental method using a relatively small amount of human blood and actual dialyzers for the simple and comprehensive evaluation of blood compatibility of hemodialysis membranes without needing to break down the actual hemodialyzers.

## Materials and methods

### Ethical approval

All subjects enrolled in this research have given their informed consent, which has been approved by the institutional committee on human research at our institution, and this protocol has been found acceptable by them (Research Ethics Committee of Asahi Kasei Medical Co., Ltd., Registration No. J15011).

### Fresh human blood

Blood sampling was performed after approval to conduct this study was obtained from the Research Ethics Committee of Asahi Kasei Medical Co., Ltd. After an explanation of the experimental object and blood collection procedure was given, informed consent was obtained from five healthy volunteers, from whom we collected 160 mL of fresh whole blood containing 1 U/mL unfractionated heparin.

### Method for rotating a hemodialyzer filled with human blood

**Fig. 1:**
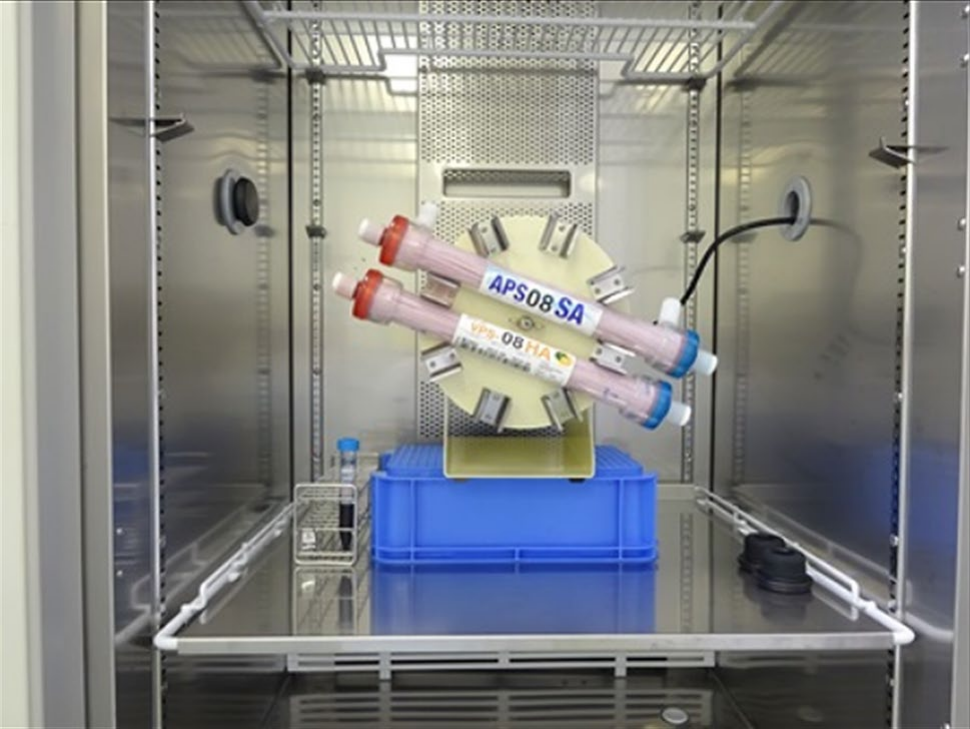
The experiment’s configuration. A polysulfone hemodialyzer and a vitamin E-bonded polysulfone hemodialyzer, both including human flesh blood, were fixed at the hooks of the rotating device and incubated while rotating at 5 rpm for 4 h at 37 °C

First, priming with 1000 mL of physiological saline at 200 mL/min was conducted on an APS™-08SA (Asahi Kasei Medical Co., Ltd.; hereinafter referred to as APS) containing PS membrane and a VPS™-08HA (Asahi Kasei Medical Co., Ltd.; hereinafter referred to as VPS) containing VE-bonded PS membrane. Then, the hemodialyzers were filled with 60 mL fresh human whole blood, while replacing the physiological saline. After the solution was removed on the dialysate side, the hemodialyzers were fixed to the rotating device (TAITEC RT-50) and incubated while rotating at 5 rpm to prevent spontaneous blood cell sedimentation for 4 h at 37 °C (Fig. 1).

### Blood analysis

#### Leucocyte and platelet

A Sysmex XT-1800i was used to measure the leukocyte and platelet count in the blood sample.

#### Thrombin–antithrombin III complex and potential antioxidant

Centrifugation of the blood samples at 3500 rpm for 10 min was carried out to obtain the plasma samples. After centrifugation, using the obtained plasma, chemiluminescent enzyme immunoassay was used to measure thrombin–antithrombin III complex (TAT), and a test kit (Test for Potential Antioxidant: PAO, Japan Institute for the Control of Aging, NIKKEN SEIL Co., Ltd.) was used to measure PAO, which corresponds to the copper reduction titer in plasma, as an indicator of the antioxidative activity of plasma.

#### Tumor necrosis factor-α, interleukin-6, interleukin-1β, interleukin-8, and interferon-γ

Centrifugation of the blood samples at 3500 rpm for 10 min was carried out to obtain the plasma samples. After centrifugation, using the obtained plasma, a test kit (Bio-plex Pro Human Cytokine Screening Panel, 48-Plex, Bio-Rad Laboratories, Inc.) was used to measure inflammatory cytokines, that is, tumor necrosis factor-α (TNF-α), interleukin (IL)-6, IL-1β, IL-8, and interferon-γ (IFN-γ).

### Quantitative analysis of total protein adsorbed on the hemodialysis membrane surface

After collection of blood from each hemodialyzer, we flushed 500 mL of physiological saline inside the hemodialysis membranes at 100 mL/min and the flushed membranes were cut into fractions of 2–3 mm. We extracted the membrane fractions in 1% sodium dodecyl sulfate/phosphate-buffered saline (−) at 1300 rpm for 4 h at room temperature. We used the bicinchoninic acid assay method (Pierce™ BCA Protein Assay Kit, Thermo Fisher Scientific Inc.) to measure the amount of total proteins adsorbed to the hemodialysis membranes.

### Quantitative analysis of fibrinogen adsorbed on the hemodialysis membrane surface

The process for the analysis of fibrinogen is the same as that for the analysis of total protein, but extraction of membrane fractions was done with 0.5% Triton-X/phosphate-buffered saline (−) at 1300 rpm for 1 h at room temperature. The competitive enzyme-linked immunosorbent assay method (Fibrinogen, Human, ELISA Kit, Assay Max, EF1040-1, Assay Pro LLC.) was used to measure the amount of fibrinogen adsorbed to the hemodialysis membranes.

### Statistical analysis

All data (from five participants) are expressed as mean ± standard deviation. For statistical analysis, to compare APS and VPS, we performed a paired *t* test. *p* values of < 0.05 and < 0.01 were used to denote statistical significance. To check the normality, the data of the plasma concentration of inflammatory cytokine were log-transformed and examined using the Shapiro–Wilk test before the paired *t* test was performed.

## Results

Table 1 shows all experimental results. Both leukocyte and platelet counts in the whole blood were significantly higher for VPS than for APS (leukocyte: *p* = 0.028; platelet: *p* = 0.015). TAT was significantly lower for VPS than for APS (*p* = 0.009), whereas PAO was significantly higher for VPS than for APS (*p* = 0.039). The plasma concentration of inflammatory cytokines tended to be lower for VPS than for APS, with significant differences for some (TNF-α: *p* = 0.005; IL-6: *p* = 0.065; IL-1*β*: *p* = 0.029; IL-8: *p* = 0.002; IFN-γ: *p* = 0.200).

**Table 1:** Experimental results of the blood compatibility of hemodialyzers

Human blood components before (Pre) and after 4 h (APS, VPS) of incubation with hemodialysis membranes at 37 °C				
	Pre	APS	VPS	*p*
Leucocyte (10^2^/μL)	55.6 ± 10.4	26.6 ± 5.3	34.2 ± 5.2	*0.028
Platelet (10^4^/μL)	18.9 ± 5.0	12.2 ± 0.8	14.5 ± 0.8	*0.015
TAT (ng/mL)	5.1 ± 3.3	29.3 ± 10.0	12.7 ± 9.9	**0.009
PAO (μmol/L)	1466.4 ± 298.5	603.7 ± 80.8	756.6 ± 138.0	*0.039
TNF-α (pg/mL)	51.3 ± 19.7	166.3 ± 122.2	60.9 ± 34.5	**0.005
IL-6 (pg/mL)	12.3 ± 4.4	18.8 ± 15.8	8.3 ± 5.0	0.065
IL-1β (pg/mL)	2.7 ± 1.7	3.6 ± 1.8	1.9 ± 1.2	*0.029
IL-8 (pg/mL)	15.6 ± 7.2	201.3 ± 158.6	43.7 ± 40.7	**0.002
IFN-γ (pg/mL)	42.6 ± 4.1	37.2 ± 8.0	30.4 ± 11.6	0.200

Quantities of total protein and fibrinogen adsorbed to hemodialysis membranes (APS, VPS) after 4 h of incubation at 37 °C			
	APS	VPS	*p*
Adsorbed protein (μg/cm^2^)	28.6 ± 3.3	23.8 ± 4.8	0.231
Adsorbed fibrinogen (μg/cm^2^)	0.071 ± 0.031	0.0067 ± 0.0037	*0.012

The numeral values are expressed as mean ± SD

*TAT* thrombin–antithrombin III complex, *PAO* potential antioxidant, *TNF-α* tumor necrosis factor-α, *IL* interleukin, *IFN-γ* interferon-γ

*p < 0.05

**p < 0.01

The amount of total proteins adsorbed to the hemodialysis membranes tended to be less for VPS than for APS (*p* = 0.231). Moreover, the amount of fibrinogen adsorbed to the hemodialysis membranes was significantly less for VPS than for APS (*p* = 0.012).

## Discussion

### Leucocyte, platelet, TAT, adsorbed protein, and adsorbed fibrinogen

Sasaki [6] conducted an  in vitro study using the miniaturized hemodialyzer of PS-UW (Kawasumi Laboratories. Inc., Minato-ku, Tokyo, Japan) and VE-coated PS-UW left in contact with human platelet-rich plasma for 30 min (*n* = 6). That study revealed a significantly lesser amount of platelets adhering to the hemodialysis membrane for VE-coated PS-UW than for PS-UW.

A prospective, open-label, nonrandomized, single-arm clinical study (14 patients) using ViE-21 (Asahi Kasei Medical Co., Ltd.), which is a hemodialyzer containing VE-bonded PS hemodialysis membranes, was conducted by Kiaii et al. [7]. Their results showed that after switching to ViE-21, leukocyte count drop during hemodialysis was inhibited, and platelet count drop after hemodialysis was also significantly inhibited. Thus, it was suggested that VE-bonded PS membranes can inhibit the adhesion of leukocytes and platelets to hemodialysis membranes.

In the present study (Table 1), we found a lesser platelet and leukocyte adhesion to the hemodialysis membrane for VPS than for APS. In addition, TAT was also significantly lesser for VPS than for APS. Moreover, a notable result was that the amount of fibrinogen adsorbed to the hemodialysis membrane was significantly lesser for VPS compared with APS. These results clearly indicate that compared with APS, VPS has better antithrombotic and anticoagulant properties, which are generally in line with the findings of Sasaki and Kiaii et al. The reason why the adsorbed protein and fibrinogen of VPS is lower than that of APS is currently unknown. A detailed analysis of the membrane surface is needed in the future.

### Potential antioxidant

Kuroda et al. [8] conducted an  in vitro study using miniaturized hemodialyzers of PS membrane and VE-bonded PS membrane (Asahi Kasei Medical Co., Ltd.), filled with heparinized fresh human blood, which were left to stand at 37 °C for 1 h (*n* = 6). The results of that study found a significantly lower plasma concentration of ox-LDL, superoxide dismutase activity, and glutathione peroxidase activity for the VE-bonded PS membrane than for the PS membrane.

A single-center, crossover study using a PS hemodialyzer (Fresenius-Kawasumi Tokyo, Japan) and a VE-bonded PS hemodialyzer (Asahi Kasei Medical Co., Ltd.) was conducted by Morimoto et al. [9]. The results suggest that during the period of treatment with VE-bonded PS hemodialyzer, plasma concentrations of ox-LDL, MDA-LDL, and asymmetric dimethylarginine in the VE-bonded PS hemodialyzer group (*n* = 16) were significantly lower than that of the PS hemodialyzer group (*n* = 15).

In the present study, the plasma level of PAO was significantly higher for VPS than for APS, as shown in Table 1. This indicates, generally in line with the findings of Kuroda et al., and Morimoto et al., that VPS has a higher antioxidative activity than APS.

### TNF-α, IL-6, IL-1β, IL-8, and IFN-γ

Sasaki [6] conducted an in vitro study using the miniaturized hemodialyzer of PS-UW (Kawasumi Laboratories. Inc.) and VE-coated PS-UW filled with the heparinized human whole blood at 37 °C, left to stand for 10 min, and the plasma concentration of granulocyte elastase was measured (*n* = 6). The results showed significantly lower concentrations of granulocyte elastase for VE-coated PS-UW than for the PS-UW.

A multicenter, crossover study was conducted by Panichi et al. [10] to compare a PS hemodialyzer with a VE-bonded PS hemodialyzer (ViE) (Asahi Kasei Medical Co., Ltd.). Their results indicate that compared with the PS group (*n* = 31), the ViE group (*n* = 31) experienced significant decreases in plasma concentrations of C-reactive protein and IL-6 after 6 months of long-term use.

Our results (Table 1) show that compared with APS, VPS had significantly lower concentrations of inflammatory cytokines (TNF-α, IL-1β, and IL-8). These results, which are generally in line with the findings of Sasaki and Panichi et al. indicate the anti-inflammatory properties of VPS.

We can, therefore, say that our experimental results obtained by new experimental method were fairly consistent with many previous findings, including clinical studies using PS type hemodialyzers (such as APS, VPS). Furthermore, our method has an advantage of an extremely simple evaluation process, with commercially available actual hemodialyzer, as employed in clinical use. As another advantage of this method, human blood compatibility can be evaluated comprehensively. Although the evaluation is performed under a condition where there is no blood flow (no shear stress), it is possible to detect the accurate blood compatibility of the dialysis membrane material itself, by eliminating any influence of factors unrelated to the hemodialysis membrane material, such as circulation conditions and circuit materials.

Our method can be applied to PS type membranes with relatively low protein adsorption. However, as a limitation, it may be difficult to apply this method to “adsorption membranes” (e.g., polymethyl methacrylate) that adsorb plasma proteins.

## Conclusion

A novel method for evaluating the blood compatibility of hemodialysis membranes, the method for rotating a hemodialyzer filled with human blood, has been established in this study. It enables a simple and comprehensive evaluation using a relatively small amount of human blood and actual PS type hemodialyzers with relatively low protein adsorption.
